# Smoky Diagnosis: Importance of Patient History in Vaping Associated Lung Injury

**DOI:** 10.7759/cureus.19596

**Published:** 2021-11-15

**Authors:** Rohit Munagala, Asad Ullah, Chinmaya Sharma, Arjun N Bhatt, Jayanth Keshavamurthy

**Affiliations:** 1 Radiology, Augusta University Medical College of Georgia, Augusta, USA; 2 Pathology, Augusta University Medical College of Georgia, Augusta, USA

**Keywords:** e-cigarettes, smoking, vaping, respiratory complications, e-cigarette or vaping use-associated lung injury (evali)

## Abstract

E-cigarette or vaping use associated lung injury (EVALI) recently became a common cause of respiratory illness. The pathophysiology of EVALI is relatively unknown, and thus the disease remains a diagnosis of exclusion. There are no specific tests or markers that exist, although there is some belief that Vitamin E acetate is strongly linked to the increase in EVALI cases. Immediate recognition of EVALI patients is critical in order to reducing severe outcomes. For these cases, the importance of a complete patient interview is emphasized and necessary for diagnosis. We present a case of a young patient presenting with hypoxic respiratory failure due to EVALI, in which diagnosis was delayed due to incomplete patient history.

## Introduction

E-cigarette or vaping use associated lung injury (EVALI) is a rapidly increasing cause of acute hypoxic respiratory failure. The number of these cases has dramatically increased since its first diagnosis in August 2019 [1]. This increase may stem from the common misconception that the use of e-cigarettes is less harmful than their organic counterparts. Based on statistics, EVALI seems to be a disease primarily limited to the United States [2]. Due to the lack of diagnostic criteria for EVALI, diagnosis is made based purely on clinical judgement. The pathophysiology of EVALI is relatively unknown; however, the leading theory is that Vitamin-E Acetate (VEA) plays a role in the lung damage associated with the disease [3]. Evidence of VEA has been seen while analyzing the bronchoalveolar lavage samples of patients presenting with EVALI [4]. Additional studies postulate that flavored additives are thought to be associated with damaging effects. The histology and imaging associated with EVALI are known to be non-specific. On pathology, any pattern of lung injury was seen, such as fibrinous pneumonitis, diffuse alveolar damage, or pneumonia accompanied by bronchiolitis. While not specific, foamy macrophages and pneumocyte vacuolization were present in many cases and were useful when assessed in a clinical context [3]. The lack of diagnostic markers in EVALI patients makes a pertinent history vital for early recognition of the disease state. With the growing number of cases of EVALI being reported in the US, a re-emphasis on the history-taking of physicians is required in order to prevent unnecessary complications. This necessity is demonstrated in this report of a young male who presented with acute respiratory distress with a delayed diagnosis of EVALI.

## Case presentation

An 18-year-old male presented to the emergency department with shortness of breath and pleuritic chest pain for the last two days. His clinical course also included bouts of non-bloody, non-bilious vomiting. His measured temperature was 103°F with a saturation of 90% on room air. The patient had recently started basic training at the local army fort when his symptoms began. He had no family history of any lung disease. He denied any use of cigarettes, marijuana, or any other drugs. There were no recent environmental exposures that would indicate interstitial pneumonitis. A general physical exam revealed a young male in acute respiratory distress. Chest X-Ray was performed, and he was diagnosed with community acquired pneumonia (CAP) and discharged on azithromycin. 

The following day, the patient presented with worsening symptoms despite treatment. Labs were significant for a white cell count of 15.8 thousand/mm3, and lactic acid of 2.5 mg/dL D-dimer was also noticed to be elevated. Vitals included tachycardia (110-120 beats/minute), tachypnea (15-25 breaths/min), and hypoxia requiring supplemental oxygen through a nasal cannula at 3L/min. His oxygen saturation (SpO2) on presentation was 80%, and there was an improvement in oxygenation. The patient had inspiratory crackles across the anterior lung base, but otherwise, the physical exam was completely normal. The patient began to have worsening somnolence and increasing tachypnea to 40 breaths/min. He required intubation and was started on ventilator settings of Tidal Volume (TV) 450 mL, the rate at 15 breaths/minute, positive end-expiratory pressure (PEEP) of 8, and fraction of inspired oxygen (FiO2) of 100%. Differential diagnosis in clinical context included pulmonary embolism, pleural effusion, pneumothorax, atelectasis, asthma exacerbation, bronchial anatomical obstruction, or complicated pneumonia. 

**Figure 1:**
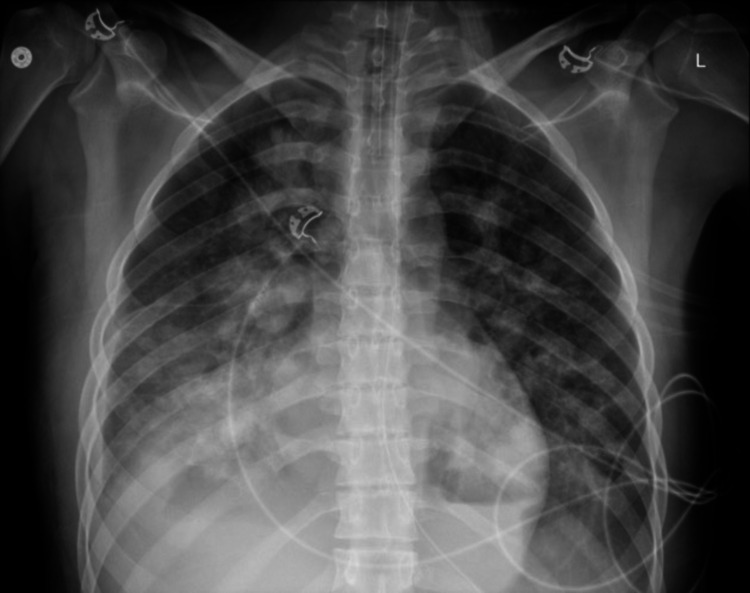
Post-intubation CXR showing severe bilateral airspace opacities with no evidence of pneumothorax or other pathology.

**Figure 2:**
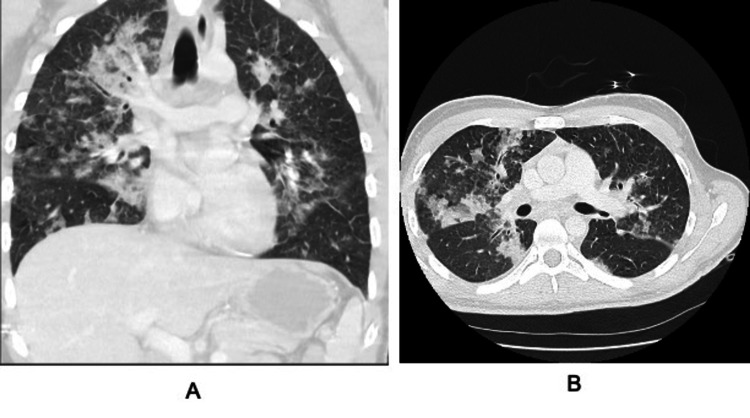
CT scans showing diffuse bilateral infiltrates with ground glass opacities and no evidence of pulmonary embolism.

**Figure 3:**
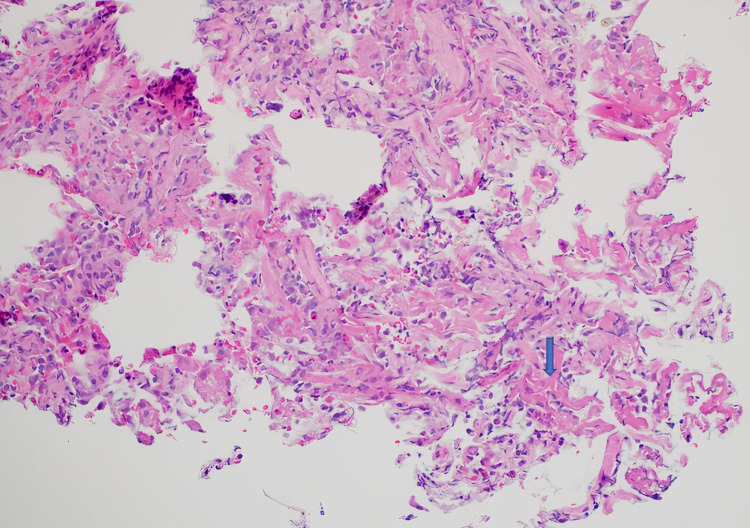
Fragments of respiratory tissue with diffuse alveolar septal thickening, abundant intra-alveolar fibrin (blue arrow) and with organizing pneumonia pattern.

Post-intubation chest X-ray (CXR) revealed nonspecific severe bilateral airspace opacities with no pneumothorax (Figure 1). Pulmonary embolism was suspected, and computed tomography (CT) showed diffuse, bilateral infiltrates with ground glass opacities. There was no evidence of pulmonary embolism (Figure 2). Discussion with the patient’s colleagues revealed that he did partake in e-cigarette use, and an e-cigarette with several liquid pods was found in his belongings. Bronchoscopy with trans-bronchial biopsy was performed and revealed fibrinous material admixed with a focal organizing pneumonia pattern (Figure 3). Bronchoalveolar lavage indicated lipid-laden macrophages. The patient was started on empiric antibiotics. No growth was seen in any cultures, and thus vancomycin and flagyl were discontinued. Due to likely EVALI, the patient was started on solumedrol IV 60 mg every six hours. Over the course of three days, he was slowly weaned from the ventilator. Upon extubating, he admitted to using one of his friend's vape pens. He was switched from IV solumedrol to prednisone 40 mg PO daily. He was transferred to the floor and discharged the next day on oral prednisone 20 mg for a planned taper. He was additionally educated on the harmful effects of e-cigarettes and was recommended to cease use immediately. 

## Discussion

Between August 2019 and January 2020, there were a total of 2602 cases of EVALI reported to the CDC, with 57 deaths being confirmed [2]. The increase in these cases brings to light the epidemic that is consuming younger populations. Most of these patients present with respiratory symptoms of cough, chest pain, and shortness of breath, but fever, chills, and weight loss are often seen as well. Gastrointestinal symptoms of abdominal pain, nausea, vomiting, and diarrhea may also occur in up to 77% of patients and may even precede the actual respiratory symptoms [3]. Physical exam may be non-contributory and diagnostic studies such as leukocytosis, erythrocyte sedimentation rate (ESR), chest X-ray, and CT all may be relatively non-specific. Histological findings include nonspecific inflammation, organizing pneumonia, and lipid-laden macrophages with fibrin deposition [5]. Lipid-laden macrophages are key to many respiratory sequelae, forming a differential of chronic smoking, fat embolism, aspiration, vaping injury, pulmonary alveolar proteinosis, or even gastroesophageal reflux disease (GERD) [6]. Thus, diagnosis is made based on clinical suspicion and proper history-taking.

The Centers for Disease Control and Prevention (CDC) recommends asking about recent use of the e-cigarette or vaping products in a confidential and non-judgmental manner [4]. Patients may be reluctant to disclose the use of such devices due to stigma as well as the illicit use of tetrahydrocannabinol (THC)/purchase from dealers. The exclusion of such information can delay management and result in poor outcomes. If product use is confirmed, the type of substances and where they were obtained from should be determined as VEA is often linked to counterfeit devices or products [7]. VEA has been linked as a likely source of lipids and causal factors in EVALI cases [7]. VEA has been identified in 94% of EVALI bronchoalveolar fluid samples [8]. VEA was not noticed in the samples of those individuals without EVALI, possibly implicating the compound in the pathophysiology of the disease. VEA may prevent the normal oxidative stress and inflammatory responses by interfering with the physiological lung function of phospholipids and surfactants of lining fluid. There is still a need for evidence-based studies to assess the complete role of VEA in EVALI [3].

Management of these patients after diagnosis should be rapid. Hospital admission is recommended for patients with saturations less than 95%. Patients often progress to severe hypoxemia and respiratory failure shortly after showing mild symptoms. All patients should receive CXR, which may show patchy infiltrates and ground-glass opacities. Patients with moderate to severe symptoms should have CT of the chest. Microbiology specimens from sputum and blood should be obtained in order to guide antibiotic management. Rapid testing for influenza should be performed. Oral or intravenous corticosteroids have been recommended, although a specific dose and duration are currently not established. Follow-up should occur after discharge in one to two weeks. Physicians should also be sure to counsel their patients on the detrimental effects of such devices and strongly discourage the use of vaping products [5].

## Conclusions

EVALI should be considered in patients presenting with acute onset of respiratory distress or respiratory failure. It is important to obtain a thorough history, including vaping or e-cigarette use. Chest imaging and bronchoscopic findings are helpful. Although the treatment is primarily supportive, the high-dose steroid can be beneficial.
